# PROPHYlactic Implantation of BIOlogic Mesh in peritonitis (PROPHYBIOM): a prospective multicentric randomized controlled trial

**DOI:** 10.1186/s13063-022-06103-4

**Published:** 2022-03-04

**Authors:** F. Coccolini, A. Tarasconi, G. L. Petracca, G. Perrone, M. Giuffrida, C. Disisto, M. Sartelli, P. Carcoforo, L. Ansaloni, F. Catena

**Affiliations:** 1 General Emergency and Trauma Surgery, Pisa University Hospital, Pisa, Italy; 2 Emergency Surgery Department, Parma University Hospital, Parma, Italy; 3 Department of Surgery, Macerata Hospital, Macerata, Italy; 4 Department of Surgery, S. Anna University Hospital and University of Ferrara, Ferrara, Italy; 5 General Surgery, Pavia University Hospital, Pavia, Italy

**Keywords:** Biological mesh, Swine dermal collagen prosthesis, Peritonitis, Incisional hernia, Emergency surgery, Acute abdomen, Laparotomy, Abdominal sonography, Randomized trial

## Abstract

**Background:**

Patients undergoing emergency surgery for peritonitis are at increased risk of abdominal wall-related complications. In patients with peritonitis, the risk of incisional hernia (IH) is extremely elevated. The evaluation of quality of life of patients with incisional hernia showed lower mean scores on physical components of health-related quality of life and body image. Furthermore, the arise of a post-operative abdominal wall complication (i.e., wound dehiscence, evisceration and IH) greatly increases morbidity and mortality rates and prolongs the hospitalization.

**Methods:**

The present study aims to evaluate the efficacy of the use of a swine dermal collagen prosthesis implanted preperitoneally as a prophylactic procedure in urgency/emergency setting against abdominal wall complications in patients operated with contaminated/infected field in peritonitis. The sample size was defined in 90 patients divided in two arms (prosthesis positioning versus normal wall abdominal closure). The follow-up will be performed at 3, 6, and 12 months after surgery. The percentage of incisional hernias, wound infections, and adverse events will be investigated by physical examination and ultrasound.

**Discussion:**

The objective is to evaluate the possibility to reduce the incisional hernia rate in patients undergoing urgent/emergent laparotomy in contaminated/infected field with peritonitis by using swine dermal collagen prosthesis preperitoneal positioning as a prophylactic procedure.

**Trial registration:**

ClinicalTrials.gov NCT04681326. Registered (retrospectively after first patient recruited) on 23 December 2020.

## SPIRIT checklist

**Table:** 

Title	PROPHYlactic Implantation of BIOlogic Mesh in peritonitis (PROPHYBIOM): a prospective multicentric randomized controlled trial
Trial registration	The trial has been registered on clinicaltrials.gov (ID number: NCT04681326) from 23 December 2020.
Protocol version	Version 2 of 6 August 2019
Funding	The trial is funded by the Italian Ministry of Health by a 2018 finalized research grant (financial years 2016-2017).
Author details	Fausto Catena is the Principal Investigator (U.O. Chirurgia d’Urgenza, University Hospital of Parma, Parma, Italy).
Name and contact information for the trial sponsor	Fausto Catena (U.O. Chirurgia d’Urgenza, University Hospital of Parma, Parma, Italy).
Role of sponsor	The trial is funded by the Italian Ministry of Health by a 2018 finalized research grant (financial years 2016-2017). The funders have had no influence on the design of the study and will not have influence on study results.

## Introduction

### Background and rationale

Patients undergoing emergency surgery for peritonitis are at increased risk of abdominal wall-related complications. In patients with peritonitis, the risk of incisional hernia (IH) is extremely elevated. The incidence of IH in patients operated with peritonitis is up to 54%, compared with an incidence of 11–26% in the general surgical population [1–3]. Moreover, up to 24.1% of patients with peritonitis undergoing emergency laparotomy may develop fascial dehiscence [4]. The evaluation of quality of life of patients with IH showed lower mean scores on physical components of health-related quality of life and body image [5]. The prophylactic mesh implantation demonstrated to reduce the incisional hernia rate in patients undergoing vascular or bariatric procedures [6–8]. However, the intraperitoneal non absorbable mesh implantation in infected fields is generally considered at least of doubtful safety because of the theoretical increased risk of chronic mesh infection and enterocutaneous fistula [9–11]. Most incisional hernias develop during the first 3 months after surgery, which represents the critical period for the healing of transected muscular and fibrous layers of the abdominal wall [12]. However, most studies recommended a long-term follow-up period of up to at least 5 years for midline abdominal incisions to determine the real incisional hernia rate [13, 14]. The midline abdominal incision is preferred in abdominal surgery, as it provides wide and rapid access compared other incisions. However, the incidence of incisional hernias is higher following midline abdominal incisions than in other abdominal incisions [15]. In emergency surgery, the midline incision in the majority of cases is a necessity. Several factors affect the process of wound healing: surgical site infection, poor surgical technique, and patient-related factors (i.e., peritonitis, old age, obesity, diabetes mellitus, nutritional deficiencies, hepatic cirrhosis, jaundice, renal impairment, malignancy, cardiac disease, chest problems, previous abdominal incisions, steroid therapy). Data about the use of biological prosthesis in infected fields are scarce and derive principally from case reports and case series [16]. However, indications about their use and usefulness in infected fields have been recently published by the Italian Biological Prosthesis Working Group (IBPWG) [16]. A previously published prospective observational study evaluated the efficacy of implantation of biological prosthesis in high risk patients in order to reduce the incidence of incisional hernia. This study suggested the efficacy of this kind of prosthesis in reducing incisional hernia rate in patients with multiple risk factors [17]. A recently published meta-analysis showed as the use of biological prosthesis in ventral hernia repair resulted in a lower infectious wound complication rate but in an similar recurrence rate. These results support the application of biological prosthesis in high risk patients [18]. One recent systematic review evaluated the positive effect on incisional hernia rate of the prophylactic mesh positioning in high risk patients [19]. No randomized trials have been published since now about the use of biological prosthesis in contaminated or infected fields. The primary endpoint is to evaluate the incisional hernia rate after laparotomy at 3–6–12 months in patients with or without the mesh. So, the rationale of the trial is to evaluate the efficacy of the use of swine dermal collagen prosthesis implanted preperitoneally as a prophylactic procedure against incisional hernia in patients operated in urgency/emergency setting in contaminated/infected fields with peritonitis. The aim of the study is to reduce the incidence of incisional hernia from 50% to 20%.

### Basic information

Swine dermal collagen prosthesis is an acellular collagenic membrane of swine origin deatigenated and naturally cross-linked. The prosthesis is latex free and free from phthalates. This system allows to eliminate the antigenic cellular component maintaining the extracellular collagenic components. These factors enhance the host tissue cells ingrowth into the prosthesis. Production and shipment of the prosthesis are performed according to the international standards EN ISO 13485:2016 and EN ISO 13485:2016.

## Methods/design

### Objective

The primary objective is:

1) Evaluate the possibility to reduce the incisional hernia rate (from 50% to 20%) in patients undergoing urgent/emergent laparotomy in contaminated/infected field with peritonitis by using swine dermal collagen prosthesis preperitoneal positioning as a prophylactic procedure.

The secondary objective is:

1) Evaluate the impact on morbidity and mortality of the systematic swine dermal collagen prosthesis preperitoneal positioning as a prophylaxis for incisional hernia in patients operated in contaminated/infected field with peritonitis.

### Ethics

The trial will be conducted in accordance with the Declaration of Helsinki and according to local and regional ethical standards. The study was approved by the Local Ethical Committee on 06 February 2020 with protocol number 506/2019/DISP/AOUPR.

### Study endpoint

The primary endpoint is to evaluate the incisional hernia rate at 3, 6, and 12 months after surgery in each group (study arm and control arm). The secondaries endpoints are to define morbidity (adverse events (AE) and serious adverse events (SAE)), surgery time, time to drain removal, length of stay in hospital, and mortality.

### Study design

This is a prospective, randomized controlled, post-marketing clinical study with medical device. The trial is proposed as multicentric with coordinator University Hospital of Parma (Emergency and Acute Care Surgery, Parma University Hospital, Parma, Italy). Now, there is only one other center that enlist patients that is University Hospital of Pisa (Emergency Surgery Unit, Pisa University Hospital, Pisa, Italy). There exists the possibility that other centers, once seen the published protocol, will ask to join the study. The eventuality will be evaluated and discussed. The number of subject to be enlisted is 90, 45 for each group (study arm and control arm). At the moment, the number of patients that are enlisted is only six. The few numbers of patients is correlate with the reduction of access in ED during COVID-19 pandemic and for the increased number of patients that is treated with open abdomen (that is an exclusion criteria for the study). In reality, the number of screened patients for the study is higher, but many are not eligible because they do not meet the eligibility criteria and others refuse to participate at the study.

### Trial schedule

**Fig. 1:**
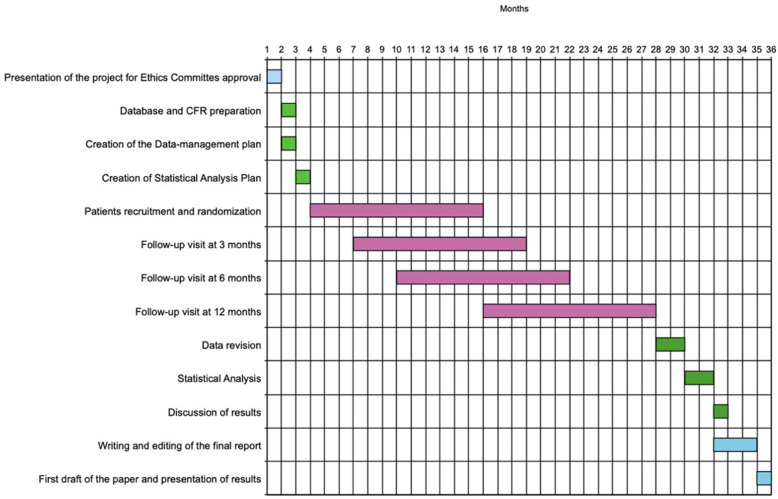
Trial time-lapse

Figure 1 is the time-lapse of the trial, from the approval by the Local Ethics Committee to the end of the study.

### Eligibility criteria

The inclusion criteria are:

- Patients aged ≥ 18 years old
- Clinical and/or laboratory and/or radiological evidence/signs of peritonitis of any origin (peritoneal reactivity, positive Blumberg sign, fever, free air/fluid in abdominal cavity, leukocytosis, increased PCR, lactic dehydrogenase (LDH), tachycardia, tachypnea, clinical or radiological evidence/suspect of bowel ischemia)
- Eventual strong suspect of possible bacterial translocation (reduction of the natural intestinal barrier against bacterial translocation, i.e., bowel ischemia, bowel overdistension, intestinal occlusion, etc.)
- Surgical indication for midline laparotomy independently from eventual previous laparotomies

#### Informed consent

All the criteria must be satisfied for included the patient in the study.

The exclusion criteria are:

- Patients aged < 18 years old
- Informed consent refusal
- No clinical and/or laboratory and/or radiological evidence/signs of peritonitis of any origin
- Surgical indication for laparotomies other than midline one
- Pregnancy

The presence of even one of these exclusion criteria does not allow the patient to participate at the study.

The subject may withdraw at will at any time. The patient may be withdrawn from the trial at the discretion of the investigator for safety concerns. If the patient withdraws or is withdrawn at any time after receiving trial product, final safety information will be obtained. Patients who are deemed during surgery not suitable included in this protocol will be withdrawn from the study. In case a subject is being prematurely withdrawn from the trial, the investigator will ensure that the procedures for the last visit are undertaken, if possible. The primary reason (adverse event, non- compliance with protocol or other) for discontinuation must be specified in the CRF. A patient withdrawn from the study will be analyzed according to evaluability of subjects for analysis.

### Patients recruitment

**Fig. 2:**
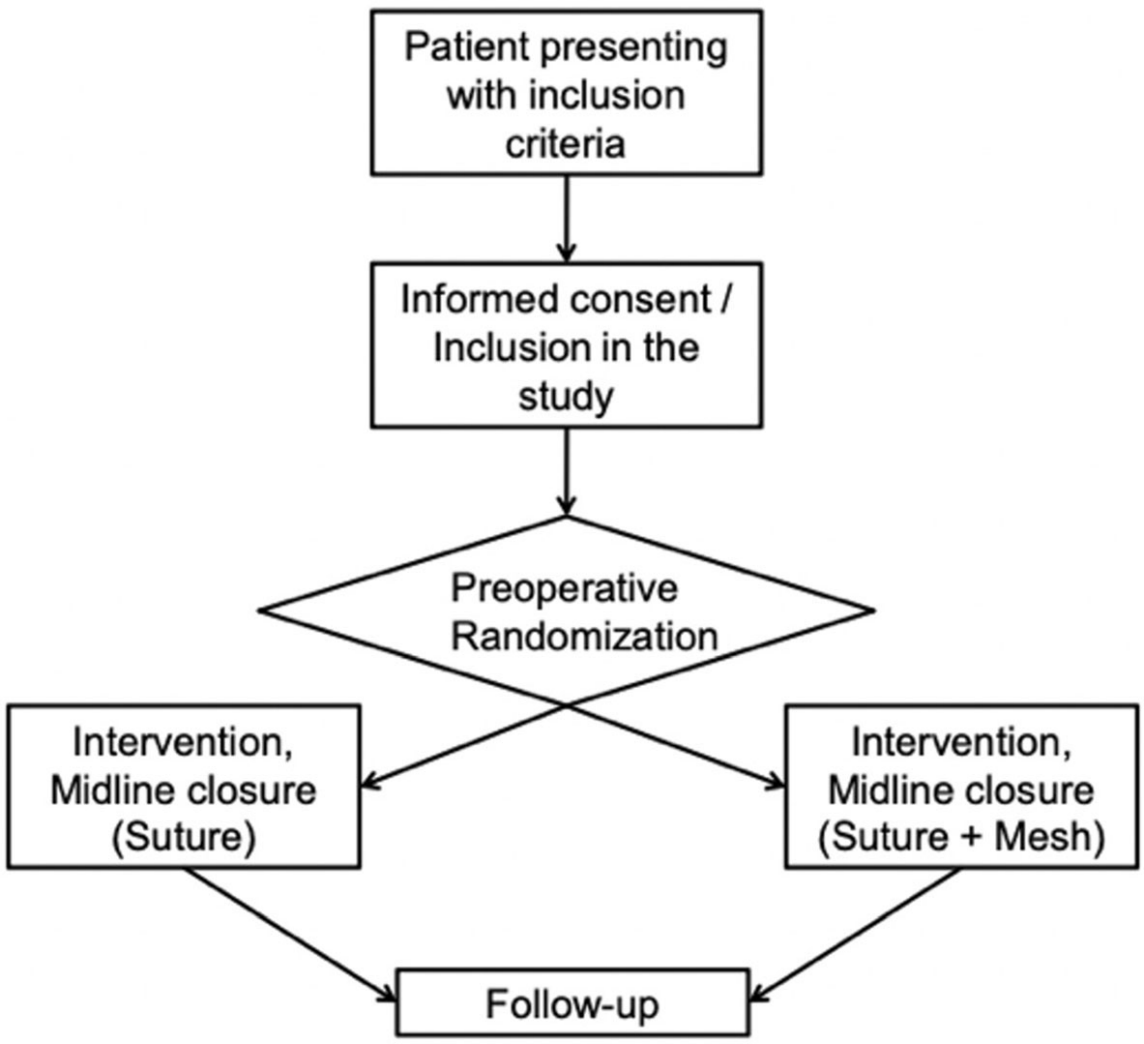
Graphical flow chart

Enrolled patients scheduled to undergo urgent/emergent laparotomy in contaminated/infected fields with peritonitis, after signing the informed consent, and before the scheduled laparotomy will be randomized (Fig. 2) to be undergone to abdominal wall closure either with mass closure technique with simple running monofilament using a long-lasting absorbable suture material or with the same technique associated to the previous retro-muscular positioning of a swine dermal collagen prosthesis (sec. Rives-Stoppa technique). The patient is identified by the surgeon that visits him/her at the arrival in ED. If the patient is eligible the same surgeon acquires consent and gives all the information about the study and the type of mesh that is used for the study. There are no ethical concerns with the randomization, because the control group will receive the standard of care treatment while the study group will receive the same treatment plus the implantation of the biological prosthesis: this prosthesis does not have reported serious side effects and it is expected to reduce the abdominal wall complication rate. If the patient allows for the study but the randomization place him in the control group, he will be treated with the standard of care (suture with running monofilament using a long-lasting absorbable suture material).

The randomization is due electronically by an online software (https://prophybiom.easycrf.cloud). The sequence of allocation has been generated by a statistician who is independent by the study. The randomization is simple and the allocation is 1:1 to each arm. The allocation sequence is accessed only when it is requested. The randomization is due electronically by the online software when the surgeon inserts the patient in the online form.

The patient is associated at an alphanumerical code that is constituted by three parts:

- First, two letters indicate the center of intervention (for example PR for University Hospital of Parma, PI for University Hospital of Pisa);
- Second, two numbers indicate the chronological sequential number of allocation (for example 01, 02, 03, etc.);
- Last, letter indicates the control group (A) or trial group (B).

So, for example, a patient that has positioning of the mesh at the University Hospital of Parma could have an alphanumerical code like “PR05B.”

### Intervention

The mesh placement will be preceded by the plane preparation. The subcutaneous tissue will be dissociated from the anterior rectum-muscles fascia to allow the positioning of the transfix stitches necessary to the mesh fixation. Successively, the retro-muscular rectum muscles plane will be prepared by the separation of the rectum muscles from the posterior rectum-muscles fascia, preparing a 5–6-cm pouch necessary to the prosthesis positioning. The mesh will be fixed with at least 8 long-lasting absorbable transfix stitched (i.e., PDS 0) placed at the cardinal and inter-cardinal points. The prosthesis will be placed with at least a 5-cm overlap. If the peritoneal plane can be sutured, a Jackson-Pratt 10 suction drain will be placed under the prosthesis. A Jackson-Pratt 10 suction drain will always be placed over the prosthesis. Anterior rectum fascia will be closed by semi-continuous monofilament suture with an intermediate-reabsorbable-time suture. Another Jackson-Pratt 10 suction drain will be placed over the anterior fascia if the subcutaneous tissue is thick. No subcutaneous suture will be performed. Skin stapler or interrupted stitches will be used to close the skin plane.

The swine dermal collagen prosthesis that is used is manufactured by MECCELLIS BIOTECH (75, Rue de Québec 17000 La Rochelle FRANCE) and is approved for use in Europe through a CE mark and is commercially available in Italy. The composition of the product is according to the product IFU. The swine dermal collagen prosthesis should be stored at room temperature in a dry environment.

Patients will be treated according to local hospital procedure. No additional costs (materials, salaries, other) due to the study will be charged to the hospital. Euroclone S.p.A. will supply all the prosthesis, as already happening for the usual clinical practice in the hospital the study is conducted in.

### Visit procedures

The randomization and the study data are collected using an online form (https://prophybiom.easycrf.cloud). The access at this form is allowed only at the participant at the study that has a username and password. Investigators who conduct follow-up assessments will not be blinded.

The study comprises of the following visits (Table 1):

**Table 1:** Time and event chart

Nomenclature	Visit 1	Visit 2	Visit 3	Visits 4, 5, and 6
	Screening	Treatment	Discharge	Follow-up
	Screening, baseline	Surgery	Discharge	Follow-up
**Informed consent**	**√**			
**Inclusion/exclusion criteria**	**√**			
**Demographic data (sex, age, weight, height, BMI)**	**√**			
**Medical history/concomitant illness**	**√**			
**Physical examination**	**√**		**√**	**√**
**ASA classification**	**√**			
**Vital signs**	**√**	**√**		
**Body weight and height**	**√**			
**ECG (12 lead)**	**√**			
**Hematology and coagulation parameters**	**√**		**√**	
**Blood chemistry**	**√**		**√**	
**Antibiotic therapy**	**√**			
**Start of LMW heparin**	**√**			
**Concomitant medication**^**a**^	**√ continuously**			
**Start and end of surgery**		**√**		
**Site and length of incision**		**√**		
**Peritonitis grade assessment**		**√**		
**Peritoneal fluid sampling for microbiological examination**		**√**		
**Contamination assessment**		**√**		
**Intestinal resection**		**√**		
**Stoma creation**		**√**		
**Surgical complications**		**√**		
**Surgical drain placement**		**√**		
**Surgery report**		**√**		
**Time of drain removal**			**√**	
**Length of stay in hospital**			**√**	
**Post-surgical complications**			**√**	**√**
**Abdominal wall ultrasound**				**√**
**Adverse events**	**√ continuously**			

^a^This includes use of any blood transfusions that should be inserted in the CRF as type (red blood cells. FFP, platelets)

• Visit 1: Screening and baseline visit: screening of patient, baseline examination, pre-surgery assessment, informed consent

• Visit 2: Treatment visit: surgery

• Visit 3: Discharge from hospital: recording of post-surgical complications

• Visits 4, 5, and 6: Post surgery follow-up visits: at 3, 6, and 12 months post-surgery. All patients will perform an abdominal wall ultrasound to document the presence or not of incisional hernia. At the last visit, the data collected is the same of the other visits. The patients are called 15 days prior to the follow-up visit to remind them of the appointment.

In case of any premature discontinuation of the trial, the patient will, if possible, be called in for a last visit. Even if the patient is not able to attend, the End of Trial Form must be completed.

### Assessments for efficacy

At discharge (visit 3), and at follow-up evaluations (visits 4, 5, and 6), the patient will be evaluated for post-surgical complications (such as hematoma/seroma, wound infection, reinterventions). Complications will be evaluated by the surgeon during surgery and at discharge. Any complications will be recorded in the CRF. If the complication leads to additional surgical interventions, it needs to be noted in the CRF. The need for blood transfusions will be noted in the CRF.

### Follow-up of adverse events

During and following a subject’s participation in a clinical trial, the investigator/institution should ensure that adequate medical care is provided to the subject for any adverse events, including clinically significant laboratory values related to the trial. The investigator/institution should inform the subject when medical care is needed for adverse event(s) of which the investigator becomes aware. The follow-up information should only include new (updated and/or additional) information that reflects the situation at the time of the investigator’s signature. All non-serious AEs classified as severe or possibly/probably related to the trial product must be followed until the subject has recovered and all queries have been resolved. However, cases of chronic conditions can be closed with an outcome of “recovering” or “not recovered.” If subjects die from another event, these cases can be closed with an outcome of “recovering” or “not recovered.” The investigator must ensure that the worst case severity and seriousness is kept consistent through the series of adverse event form and related adverse event follow-up form(s). The investigator must forward follow-up information on non-serious AEs on the adverse event follow-up form. All serious AEs must be followed until the outcome of the event is recovered, recovered with sequelae, or fatal and until all queries have been resolved. For cases of chronic conditions and cancer or if the subject dies from another event, follow-up until the outcome categories are “recovered,” “recovered with sequelae,” or “fatal” is not required, as these cases can be closed with an outcome of “recovering” or “not recovered.” There are some adverse events that are expected for the population of the study like allergic reaction to the mesh, infection of the mesh, and migration of the mesh.

### Sample size

No previous data exist on the efficacy of swine dermal collagen prosthesis in preventing incisional hernia in peritonitis patients. The following published data will be used in our sample size calculation:

- “The risk of incisional hernia in patients with peritonitis is elevated, with an incidence of up to 54%, compared with an incidence of 11–26% in general surgical population” [1–3, 20].
- “The result of this pooled analysis suggests a benefit to prophylactic mesh placement during laparotomy closure in high-risk patients with a significantly reduced incidence of incisional hernia without any significant differences in seroma formation and wound infection rate” [19].
- “The use of biologic mesh for ventral hernia repair results in less infectious wound complications but similar recurrence rate compared with non-biologic mesh” [18].

The sample size has been calculated using statpages.org (proportion difference power/sample size calculation). The sample size was defined in 90 patients (45 in each arm).

### Statistical analysis

Only the data of participants who complete the follow-up will be considered. Descriptive analysis will be performed for all pre-operative, operative, and follow-up information. For categorical variables, we will use chi-square test, for non-categorical one the *t*-test. The primary endpoint, % of patients presenting incisional hernia at 3, 6, and 12 months, will be analyzed between treatment groups using a logistic regression model, presenting odds ratio comparisons of the two. Surgery time, time to drain removal and length of stay in hospital will be analyzed between treatment groups using Student’s *T* test if data are normally distributed and with Mann-Whitney test if data are not normally distributed. The distribution of data will be evaluated with the Shapiro-Francia test. The number of patients who died will be analyzed between treatment groups using a chi-square test.

All statistical comparisons will be based on two sided tests with a 5% significance level.

### Data management

Data management is the responsibility of the principal investigator. All data relating to the various phases of the study will be collected in the appropriate electronic data collection form provided by an external company (m:gnu, Consultancy-Web Architecture-Management). The patients for whom the data is collected are cataloged with an identification code, which in the electronic version would translate into an alphanumeric code generated automatically by the system, thus avoiding the recording of personal data; in this way, the patient is de facto anonymized, and the data collected could not be considered “sensitive,” because they are not associated with an identifiable person. The cloud platforms that will be used are all GDPR compliant. The access at the anonymous data is permitted only at the principal investigator. The subject will be identified by subject number. Appropriate measures such as encryption or deletion will be enforced to protect the identity of human subjects in all presentations and publications as required by local/regional/national requirements. The data is monitoring by the principal investigator of the leader center of the study. There is a monitoring committee that monitors the data collection and study progress. This committee is the Unit of Clinical Research and Epidemiology of University Hospital of Parma. The monitoring committee monitors the data every 6 months. Statisticians, medical doctors specialized in epidemiology, and data managers are part of the monitoring committee.

When all 90 patients have been entered in the data base, data quality will be ensured and a data base release conducted.

## Discussion

To date, no randomized clinical trials have examined the efficacy of biological mesh in contaminated field, and no previous data exist on the efficacy of swine dermal collagen prosthesis in preventing incisional hernia in peritonitis patients.

Our randomized controlled trials have the aim to evaluate the impact of biological prosthesis in contaminated field, reduce the incidence of incisional hernia from 20 to 50%, and determine the impact on morbidity and mortality of the systematic swine dermal collagen prosthesis preperitoneal positioning.

The result of our study will increase the knowledge about swine dermal collagen prosthesis in peritonitis patients. The long follow-up of over 36 months would permit to understand the rate of incisional hernia in the medium-long term by comparing the patients treated with the biological prosthesis and those on which traditional treatment was performed. If the use of the swine biological prosthesis will prove to be an advantage (or disadvantage) in terms of reducing early and late post-operative complications (wound infections, seroma, wound dehiscence, bleeding, incisional hernia, re-surgery), it will probably be possible to draw up more precise guidelines in case of median laparotomies during surgery for peritonitis. In fact, the absence of clear indications on the approach in case of laparotomies on infected fields leads the surgeon to have random and different behaviors from case to case without determine the cause of possible post-operative complications.

Moreover, surgery performed in urgency for peritonitis is accompanied by a percentage of complications that are certainly higher than elective surgery. Trying to understand if it is possible to reduce the percentage of complications due to incisional hernias could also help to reduce the direct and indirect costs associated with surgery. Thus, studies that are aimed to determinate if it is possible are needed. The results of this trial will support a tangible decision-making process for choosing appropriate technical approach in abdominal parietal synthesis in peritonitis.

## Trial status

The trial was the winner of a grant from the Italian Ministry of Health for finalized research in 2018 (financial years 2016–2017) with project code RF-2018-12368001. This study in in phase IV. Patient recruitment has begun on 1 December 2020 and will end after 36 months on 1 December 2023.

## Data Availability

The data will be stored on an electronic database of University Hospital of Parma. The principal investigator will oversee the management of the final trial dataset. The study result will be used for oral presentation, publications, and seminars.
